# Inferior Vena Cava Agenesis, a Forgotten Etiology of Deep Vein Thrombosis in Young Patients: A Case Report

**DOI:** 10.7759/cureus.64089

**Published:** 2024-07-08

**Authors:** Maria Inês Matos, Carlos Grijó, Claudemira Pinto, Adriana Costa Moreira, Marta Patacho

**Affiliations:** 1 Internal Medicine, Centro Hospitalar Universitário de São João, Porto, PRT; 2 Radiology, Centro Hospitalar Universitário de São João, Porto, PRT

**Keywords:** deep vein thrombosis, inferior vena cava, congenital malformations, vascular malformations, case report

## Abstract

Agenesis of the inferior vena cava (IVC) is a rare congenital anomaly that is associated with the development of extensive collateral circulation with the aim of compensating for the inadequate return of blood to the right ventricle. This collateral circulation predisposes to the emergence of venous hypertension with stasis and thrombus formation. Most cases are asymptomatic and are diagnosed incidentally. We report the case of a 28-year-old man who presented with bilateral deep vein thrombosis (DVT) as the first manifestation of agenesis of the IVC. We decided to maintain anticoagulation for an indefinite period of time after a multidisciplinary discussion. IVC agenesis should be considered a cause of DVT in young men, with bilateral and proximal thrombosis and without other risk factors. The rarity of the condition makes its therapeutic approach complex.

## Introduction

The exact incidence of congenital malformations of the inferior vena cava (IVC) has not been established, but it is estimated that it occurs in around 0.3% of the population [1]. Deep vein thrombosis (DVT) affects around 1/1000 people per year and is more prevalent in older age groups [2,3]. IVC agenesis is a rare congenital anomaly, with an estimated prevalence of 0.0005-1% of the population. Embryonic malformation is the most commonly accepted pathophysiological mechanism, although some authors propose intrauterine or perinatal thrombosis as another potential mechanism [4]. The majority of cases are asymptomatic and are diagnosed incidentally. Despite this, IVC agenesis appears to increase the risk of DVT and its recurrence [5].

We present the case of a patient with proximal bilateral DVT associated with IVC agenesis. The patient provided written informed consent for the publication of this article and images. This article was previously presented as a meeting abstract at the 21st European Congress of Internal Medicine 2023.

## Case presentation

A 28-year-old man, a non-smoker with no relevant medical history and no usual medication, was admitted to the ED due to pain and bilateral edema of the lower limbs, which had been evolving for five days. One month earlier, he experienced bilateral lower back pain with mechanical characteristics and functional limitations. Lumbar MRI showed no significant osteoarticular pathology. The pain decreased with analgesia, but he still felt the need to suspend major physical activity. The patient denied recent trauma, surgeries, or immobilization, as well as a family history of venous thromboembolism or thrombophilias. He also denied intense physical activity in the days before the onset of pain.

**Figure 1:**
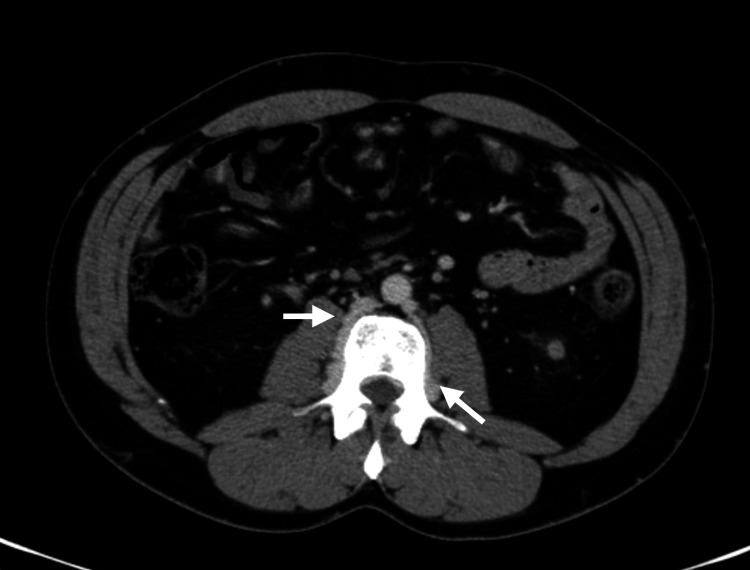
Congenital absence of the IVC with collateral circulation through prominent lumbar veins (white arrows) IVC, inferior vena cava

**Figure 2:**
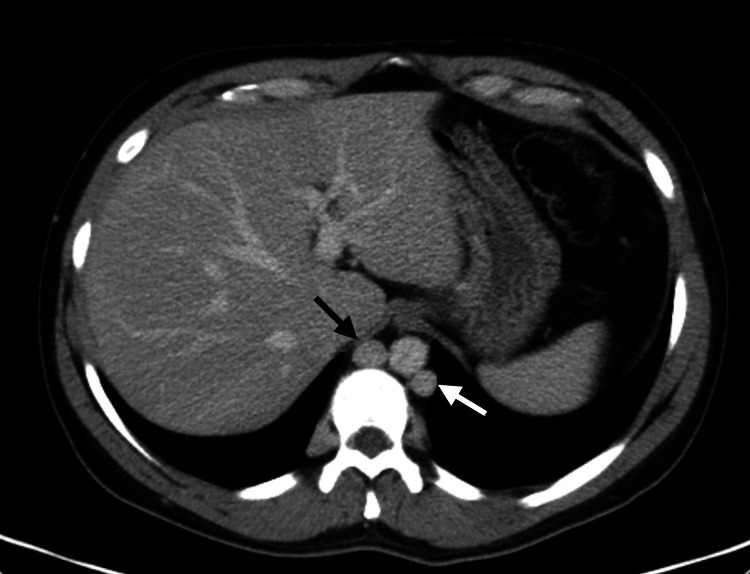
Marked prominence of the azygos (black arrow) and hemiazygos (white arrow) veins due to drainage of the collateral circulation

**Figure 3:**
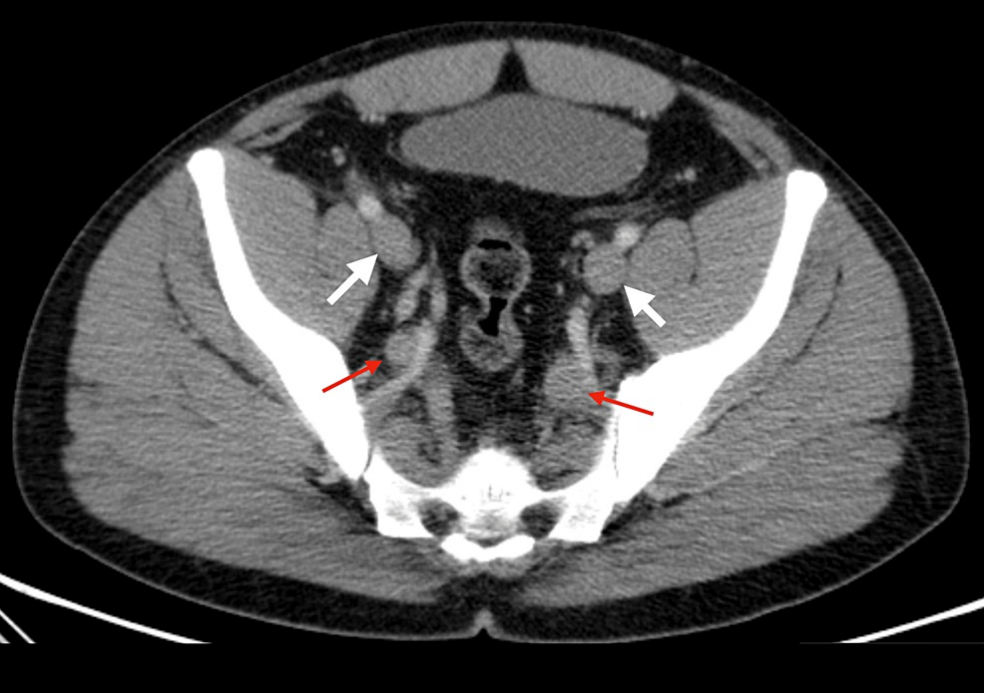
Both the internal iliac veins (red arrows) and external iliac veins (white arrows) exhibit increased caliber and are not filled with contrast, consistent with recent thrombosis

In the ED, the patient was normotensive, apyretic, and eupneic, with edema, erythema, and pain in both lower limbs, along with varicose veins. There was no evidence of collateral circulation in the chest or abdomen. Blood tests showed an increase in D-dimers without other major changes. An abdominopelvic CT scan was performed, revealing agenesis of the IVC (Figure 1), replaced by a prominent collateral network, increased circulation in the azygos and hemiazygos systems (Figure 2), renal and lumbar veins, and thrombosis of the internal and external iliac veins and femoral veins bilaterally (Figure 3). No other malformations were detected.

The transthoracic echocardiogram excluded the presence of cardiac malformations. The prothrombotic study was negative, and the presence of malignant neoplasia was excluded. Anticoagulation with unfractionated heparin was started and later switched to warfarin.

**Figure 4:**
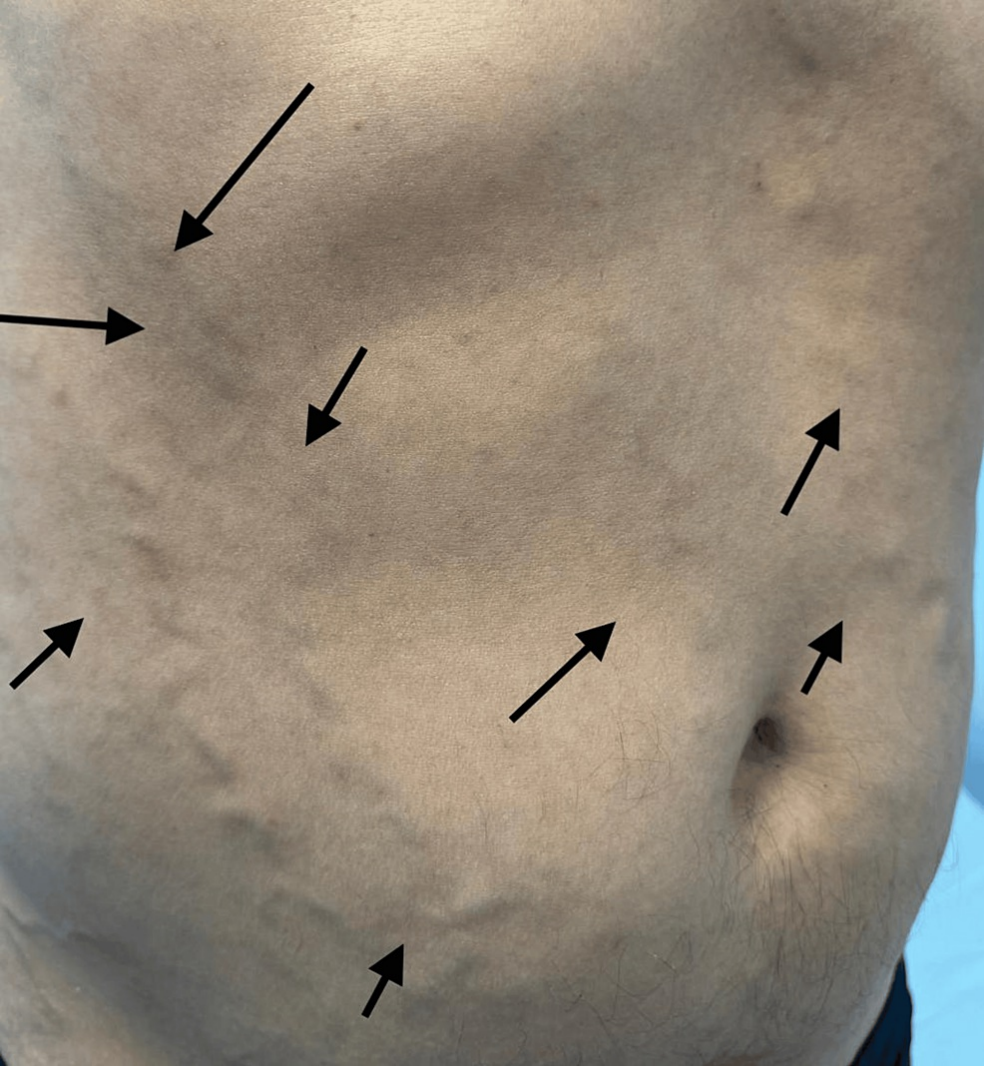
Exuberant external collateral circulation of the abdomen (black arrows)

**Figure 5:**
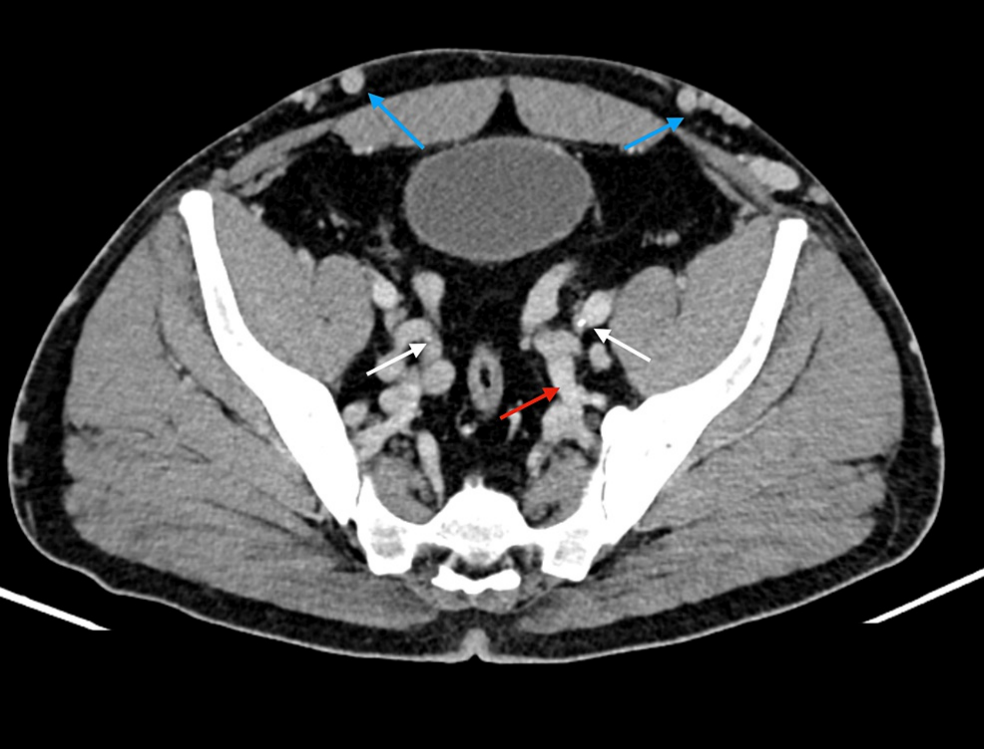
The internal and external iliac veins now exhibit filiform caliber (white arrows) without contrast filling, indicative of chronic thrombosis. Extensive deep pelvic collateral circulation (red arrows) and circulation through superficial vessels of the abdominal wall (blue arrows), namely the epigastric vessels, are observed

**Figure 6:**
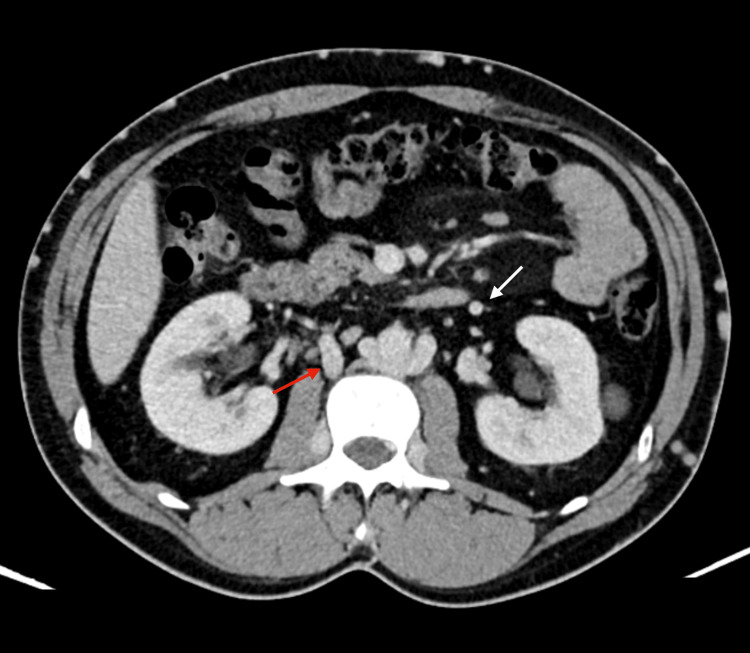
Collateral circulation is also evident through the gonadal veins (white arrow), renal veins (red arrow), and lumbar veins

**Figure 7:**
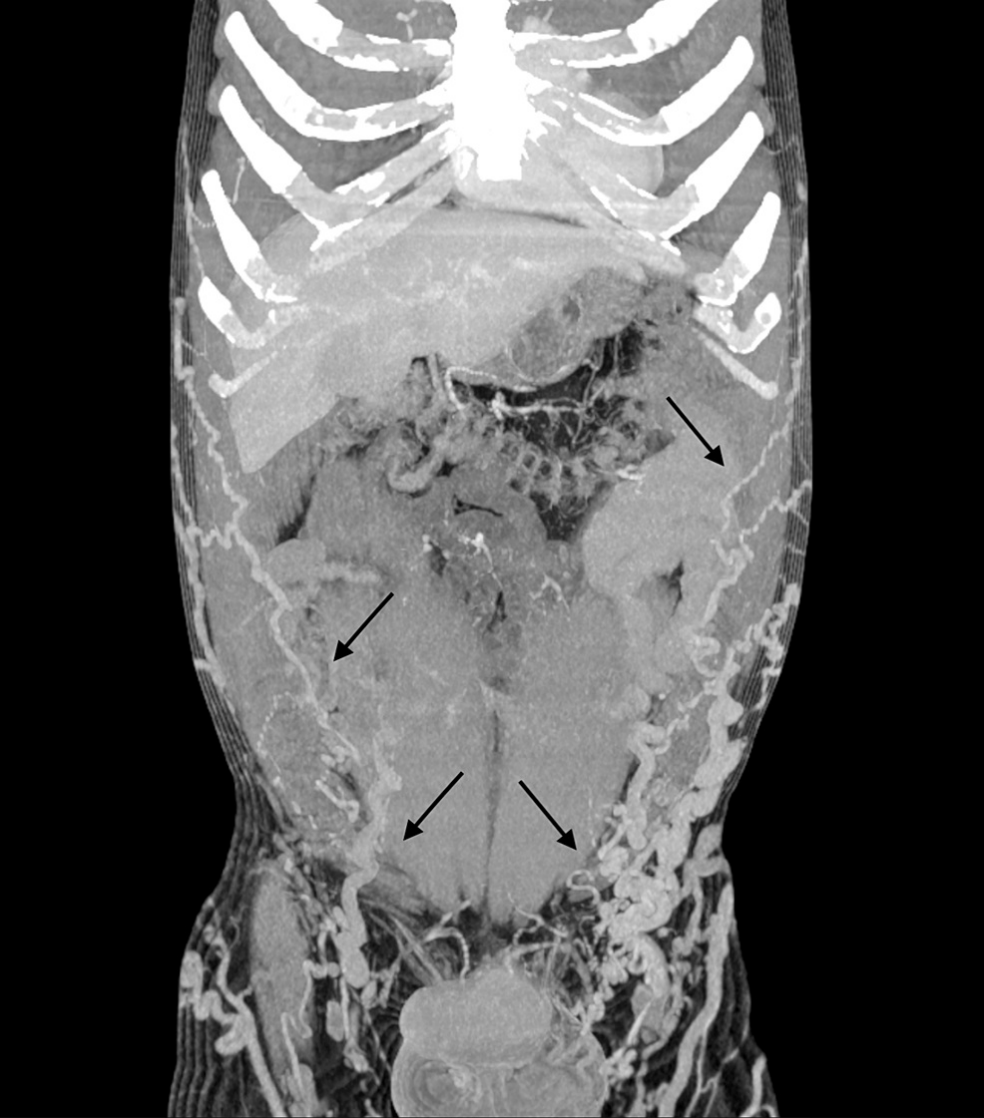
MIP reconstruction in the coronal plane showing the extensive superficial collateral circulation (black arrows) MIP, maximum intensity projection

**Figure 8:**
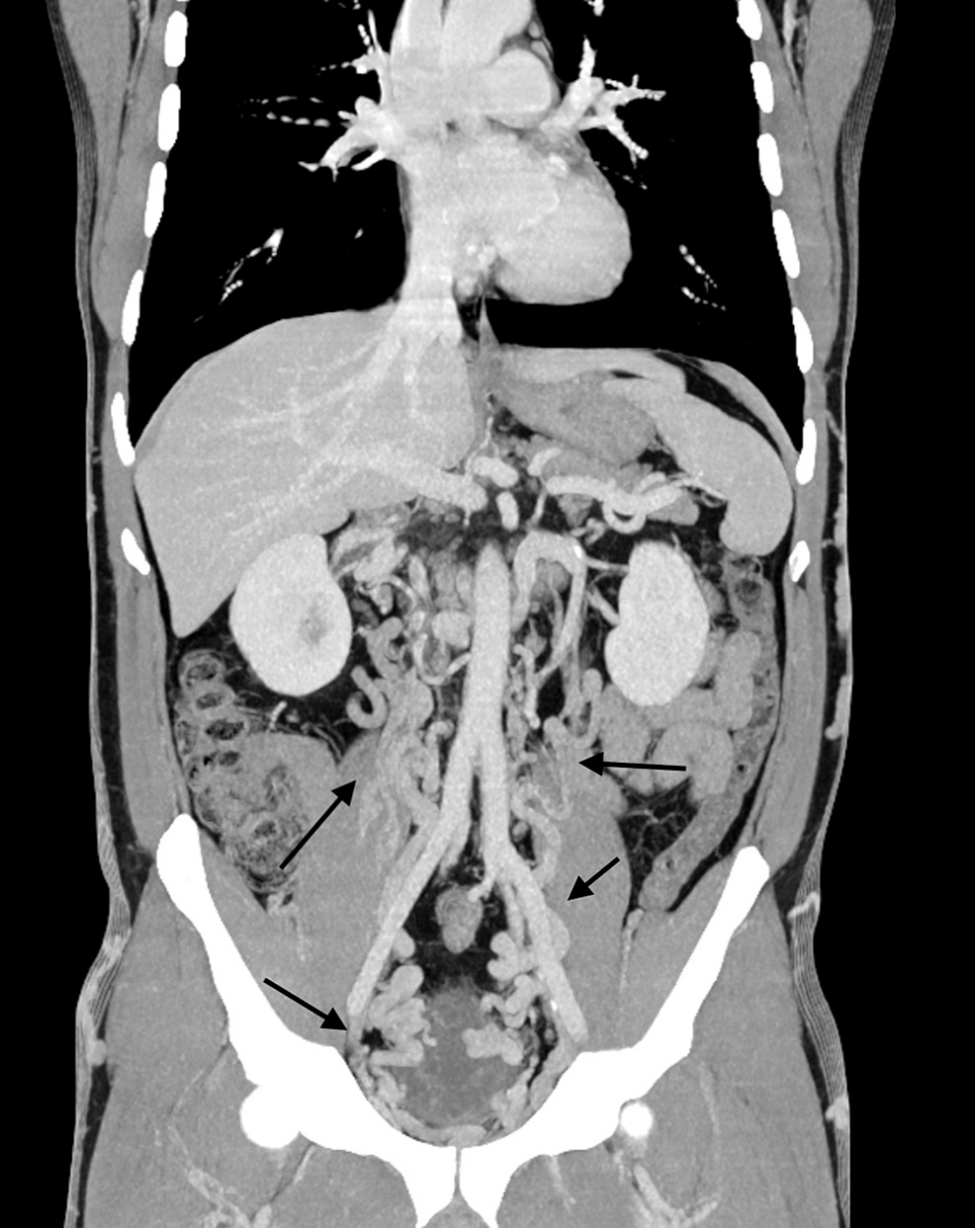
MIP reconstruction in the coronal plane showing the extensive intra-abdominal collateral circulation (black arrows) MIP, maximum intensity projection

The patient continued follow-up at the internal medicine consultation, showing improvement in edema. However, he was diagnosed with post-thrombotic syndrome, experiencing predominant pain in the lower limbs and lower back, with a Villalta score of 12 points. He developed exuberant external collateral circulation (Figure 4) and increased internal collateral circulation (Figures 5-8), predominantly in the pelvic, inguinal, azygos, and hemiazygos systems.

After confirming no new thrombotic events and no significant hemorrhagic events, following multidisciplinary discussion and consultation with the patient, a decision was made to transition anticoagulation to edoxaban and maintain it indefinitely.

## Discussion

The development of the venous system during embryogenesis is a complex process that occurs between the fourth and eighth weeks of gestation and is dependent on the fusion, regression, and anastomosis of three pairs of vessels: the posterior cardinal, subcardinal, and supracardinal veins. The final IVC is segmented into four parts: hepatic and suprahepatic segments arise from the right vitelline vein; suprarenal, which arises from the right subcardinal vein; renal, derived from the right suprasubcardinal anastomosis; and infrarenal, which arises from the right supracardinal vein [1]. Embryonic malformation is probably the most common pathophysiological mechanism of IVC agenesis [4]. In these cases, extensive collateral circulation develops in the azygos, hemiazygos, and lumbar systems, with the aim of compensating for the inadequate return of blood to the right heart [6].

The anomalies and variations in the IVC anatomy, without other associated malformations, occur in around 0.3% of the population [1]. Although IVC malformations are rare, they are diagnosed in almost 5% of patients under 30 years of age with spontaneous DVT [5]; therefore, IVC anomalies are considered, in themselves, important risk factors for DVT. Despite this, pulmonary thromboembolism is uncommon in these patients. This can be explained by the fact that the azygos/hemiazygos system, which is responsible for venous return to the pulmonary circulation, has a smaller caliber than the IVC, which could act as a filter for major embolisms [4].

Most cases are asymptomatic and are diagnosed incidentally, during abdominal surgery, imaging exams, or electrophysiological procedures [6]. A systematic review showed that IVC agenesis is more common in men, with a mean age of 27.9 ± 18 years, with the majority of patients presenting with DVT (mainly of the iliac veins), and the most common symptoms being pain and edema in the lower limbs [7]. A review of cases concluded that DVT associated with IVC agenesis usually occurs after a precipitating event, mainly intense physical exercise. This is probably associated with the inability of venous collaterals to respond to increased blood flow during major physical activity, with consequent venous stasis and thrombosis [4]. CT or MRI are the ideal imaging methods for diagnosing IVC malformations [8].

There are currently no guidelines or recommendations for the ideal treatment for these patients [9]. Treatment is mainly conservative, focusing on preventing venous stasis, clot formation, and recurrence. The basis of treatment for these patients is anticoagulation with heparin, vitamin K antagonists, or direct oral anticoagulants. In addition, it is important to avoid high-intensity physical exercise, promote the use of elastic stockings, elevate the lower limbs, and optimize the control of other risk factors for DVT. The duration of anticoagulation is a controversial topic in this population: in a retrospective observational study with nine patients with IVC agenesis and DVT, followed for 78 months, no major bleeding events occurred in anticoagulated patients, and DVT recurrence only occurred in those who discontinued anticoagulation [10]; in another retrospective observational study with six patients with the same characteristics, in whom anticoagulation was suspended after 35 months (on average), only one had a recurrence of DVT [11]. Despite this, the majority of case reports recommend anticoagulation indefinitely if there are no contraindications, taking into account the risk of DVT recurrence in these populations that have a non-modifiable risk factor [8].

Post-thrombotic syndrome is an important problem, and it constitutes the main cause of morbidity in this population. A retrospective observational study, which included nine patients with DVT and IVC agenesis, reported an incidence of post-thrombotic syndrome of 55.6% [10]; another study reported an increased incidence of severe post-thrombotic syndrome in patients with DVT and IVC agenesis [11].

The patient described presents a rare diagnosis, which was not considered in the initial differential diagnosis of DVT and which provided a learning moment for the team. Furthermore, treatment is a challenge because the literature is ambiguous and implies decisions that are not based on randomized studies or quality evidence, meaning that multidisciplinary decisions become extremely relevant. Finally, the post-thrombotic syndrome has been a complex entity to manage, as has the low back pain that we associate with venous hypertension in the lumbar circulation.

## Conclusions

This case reinforces the importance of considering IVC agenesis as a cause of DVT, especially in a particular profile of patients: men, young, with bilateral and proximal thrombosis, and without other risk factors. The absence of precipitating factors or risk factors should encourage a thorough investigation of vascular anomalies in this group of patients. The rarity of the condition makes its therapeutic approach complex, with multidisciplinary discussion being essential. A special focus should be given to the prevention of venous stasis, clot formation, and recurrence. Maintaining anticoagulation indefinitely seems to be the most consensual decision, mainly due to the risk of DVT recurrence. Post-thrombotic syndrome and extensive collateral circulation are important complications of this disease and are associated with a loss of quality of life in this patient group.
